# P-281. Risk Factors for Treatment Failure in Infections Caused by Carbapenem-Resistant Enterobacterales (CRE)

**DOI:** 10.1093/ofid/ofae631.484

**Published:** 2025-01-29

**Authors:** Michelle Ting, Mycah Martens, Haya Albazzaz, Christine Wolesensky, Emir Kobic

**Affiliations:** Banner University Medical Center Phoenix, Phoenix, Arizona; Banner University Medical Center Phoenix, Phoenix, Arizona; Banner University Medical Center Phoenix, Phoenix, Arizona; Banner University Medical Center Phoenix, Phoenix, Arizona; Banner University Medical Center Phoenix, Phoenix, Arizona

## Abstract

**Background:**

Multi-drug resistant organisms (MDROs) are a rising global public health threat. During the COVID-19 pandemic, there was a surge in New Delhi Metallo-Beta-Lactamases (NDM) amidst increasing prevalence of Carbapenem-Resistant Enterobacterales (CRE) infections in Maricopa County, Arizona. The objective of this study was to investigate risk factors associated with CRE treatment failure by measuring the composite of 30-day mortality and 30-day microbiologic recurrence.

**Table 1.**
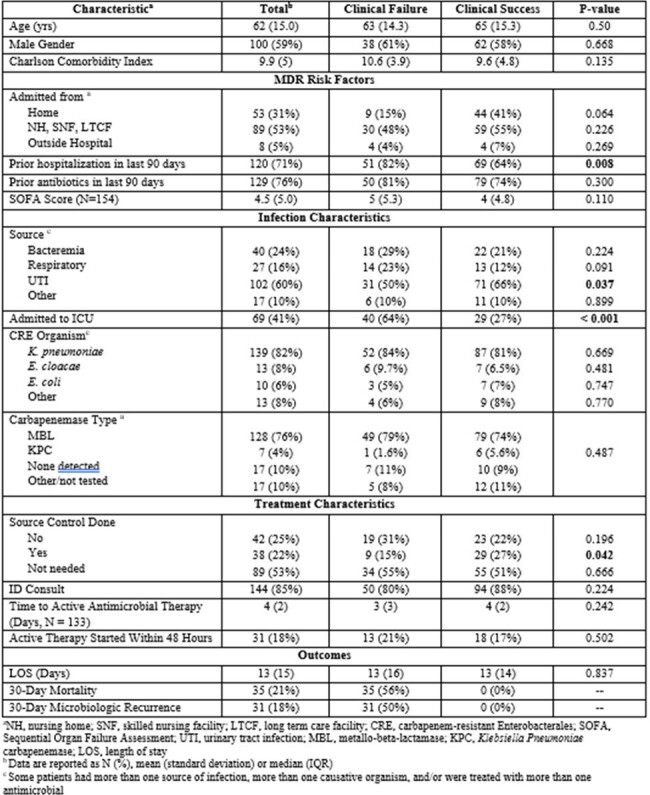
Patient Characteristics

**Methods:**

A multicenter observational retrospective chart review of a large health system’s 31 acute care facilities was performed for adult patients with infections caused by an organism in the Enterobacterales group with resistance to at least one carbapenem between January 1st, 2023 and July 31st, 2023. Key exclusion criteria were pregnancy and any patients with prior positive cultures with CRE within 60 days (only the first instance was included). Data was obtained via reports provided by pharmacy analytics.

**Table 1.**
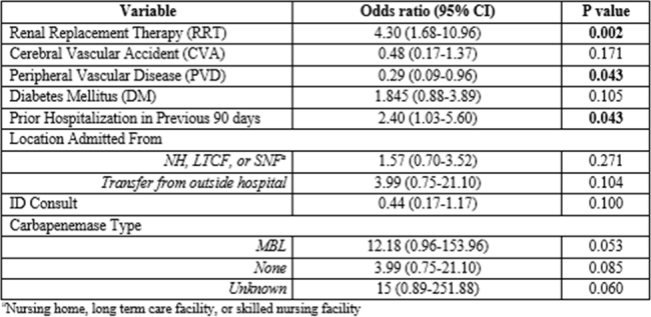
Multivariable Logistic Regression, Predictors of Clinical Failure

**Results:**

Of 169 included patients, 21% faced 30-day mortality, while 18% encountered microbiologic recurrence within 30 days. The majority (53%) of patients were admitted from assisted living facilities. The most common organism was *Klebsiella pneumoniae* occurring in 82% of cases and NDMs were the most prevalent carbapenemase, occurring in 76% of patients; urine was the source of infection in 60% of patients. Our multivariable logistic regression identified several significant risk factors associated with CRE treatment failure, including receipt of renal replacement therapy (RRT), prior diagnosis of peripheral vascular disease (PVD), and prior hospitalization within 90 days of index culture. Patients infected with an MBL producer and those not being seen by ID consult also trended towards worse outcomes.

**Conclusion:**

Our study highlights the concerning prevalence of *Klebsiella pneumoniae* infections with metallo-beta lactamases in Maricopa County Arizona, along with clinically significant risk factors contributing to treatment failure against CREs. Notably, the majority of patients arrived from a healthcare related facility, underscoring the importance of implementing best infection prevention and antimicrobial stewardship practices in these settings.

**Disclosures:**

**Emir Kobic, BCIDP**, Shionogi: Grant/Research Support|Shionogi: Speaker Bureau

